# Dentists’ Instruments

**Published:** 1883-08

**Authors:** 


### DENTISTS' INSTRUMENTS.

To protect steel instruments from rust a thin film of mercurial ointment should be spread upon them.—*Progrès Dentaire.*

